# Insights into the lifecycle of *Cryptosporidium* and compounds targeting developmental stages

**DOI:** 10.1016/j.mib.2025.102703

**Published:** 2026-01-14

**Authors:** Zoë Reynolds, Sumiti Vinayak

**Affiliations:** Department of Pathobiology, College of Veterinary Medicine, University of Illinois at Urbana-Champaign, Urbana, IL 61802, USA

## Abstract

The intestinal protozoan parasite, *Cryptosporidium*, is a leading cause of diarrhea-associated illness and death in young children, immunocompromised individuals, and neonatal ruminant animals. This apicomplexan parasite completes its entire lifecycle within a single host, involving a timely and coordinated progression through asexual and sexual developmental stages. With no fully effective drugs or vaccines available, a deeper understanding of the parasite’s lifecycle stages is crucial for identifying new molecular targets for disease intervention. In this review, we discuss recent advances in understanding the *Cryptosporidium* developmental lifecycle, stage-specific gene expression, and the role of parasite proteins in invasion, asexual proliferation, and sexual stages. We also discuss the lifecycle stages targeted by a few highly effective anticryptosporidial compounds.

## Introduction

The protozoan parasite *Cryptosporidium* is a major cause of diarrheal disease and death among children under five years in low- and middle-income countries [1–4]. Repeated episodes of cryptosporidiosis are associated with chronic malnutrition, growth stunting, and developmental defects in young children [3,5–7]. *Cryptosporidium* species (*C. parvum* and *C. hominis*) are responsible for frequent global outbreaks caused by contamination of drinking and recreational water facilities with oocysts, as well as contact with infected animals [8,9]. *Cryptosporidium parvum* infects young ruminant animals (neonatal calves, young lambs, and goat kids) and humans, while *C. hominis* infection is limited to humans. Currently, no fully effective drugs exist to treat human or animal cryptosporidiosis or to eliminate oocyst shedding. The only US Food and Drug Administration (FDA)-approved drug, nitazoxanide, has poor efficacy in children and immunocompromised individuals, and there is no vaccine to prevent cryptosporidiosis in humans [6,10–12]. *Cryptosporidium* completes its entire lifecycle (including both asexual and sexual stages) within a single host and infects only the intestinal epithelial cells (enterocytes) of the small intestine. The development of CRISPR/Cas9 gene editing and mouse models of infection has advanced the field by enabling (i) the discovery of new aspects of the *Cryptosporidium* developmental cycle, (ii) the study of parasite gene functions, (iii) insights into host-parasite interactions, (iv) testing the effectiveness of new anticryptosporidial drugs, and (v) validation of drug targets [13–18]. The past decade has seen significant progress in the field, including the expansion of the genetic toolbox and the development of new intestinal organoids and air–liquid interface culture systems for growing the parasite *in vitro* [19–21]. For cell biological studies and phenotypic characterization of *Cryptosporidium* gene knockouts, researchers rely on a human ileocecal adenocarcinoma cell line (HCT-8) that is easy to grow and maintain in the laboratory. Infected HCT-8 cultures have been used for fixed- and live-imaging studies to understand the *Cryptosporidium* lifecycle and to generate multiple gene-expression datasets across different stages of parasite development. Although *Cryptosporidium* undergoes both asexual and sexual developmental stages in HCT-8 cells, the culture stalls at the fertilization stage with no new oocyst formation [22]. However, this block in fertilization is overcome in mice and organoid cultures, leading to oocyst production.

**Figure 1:**
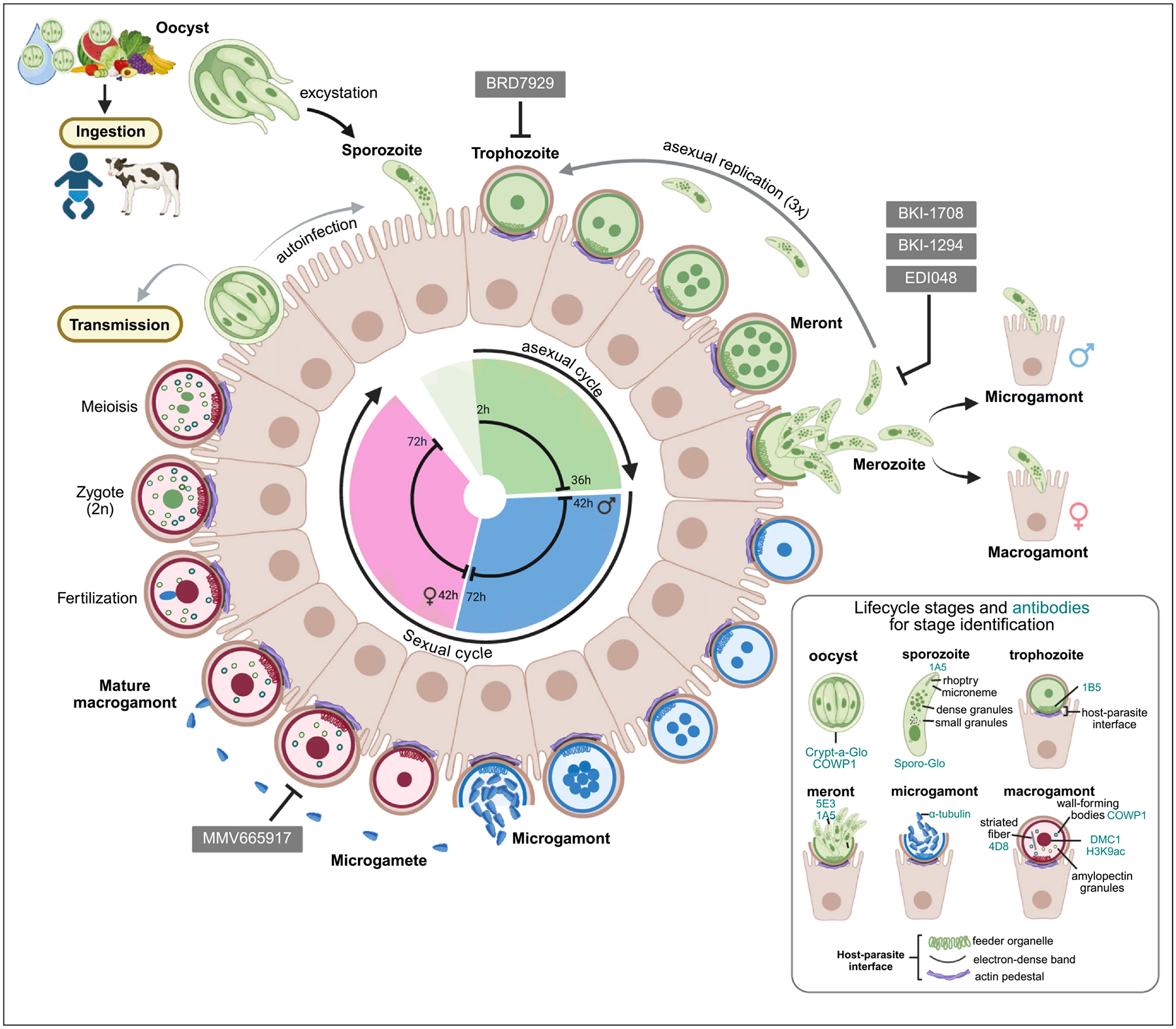
Lifecycle of *Cryptosporidium* showing the asexual and sexual developmental stages and timing of progression for each stage. We also show some antibodies (in teal) in the panel used by researchers to identify the different parasite lifecycle stages in immunofluorescence assays and imaging. Fluorescein-conjugated *Vicia villosa* lectin (VVL) is used as a general parasite stain for N-acetylgalactosamine found at the parasite membrane and stains almost all stages. Crypt-a-Glo and Sporo-Glo stain oocysts and sporozoites, respectively, and all three are commercially available. The COWP1 antibody also stains oocysts [77,82,83]. The 1A5 antibody stains the apical end of sporozoites and mature merozoites [82]. Trophozoites are identified by their single nucleus and monoclonal antibody 1B5, which stains the feeder organelle [82]. Meronts can be visualized by multiple nuclei, as well as by the 5E3 monoclonal antibody, which marks the apical end of merozoites [82]. The α-tubulin antibody marks mature microgamonts ready to egress, as well as egressed male gametes, by staining the microtubules running parallel to their bullet-shaped nuclei. Although no antibodies are available to identify each stage of microgamont development, tracking changes in nuclear morphology has proven useful [22,70]. To visualize macrogamonts in culture, H3K9Ac, DMC1, COWP1, and 4D8 are used. The histone H3 acetylated at lysine 9 (H3K9Ac) antibody labels accumulated histones in the macrogamonts, since the female nucleus is difficult to observe with nuclear stains such as 4,6-diamidino-2-phenylindole (DAPI) or Hoechst [17,22]. The DMC1 antibody stains nuclei of macrogamonts from 42 to 72 hours post-infection of HCT-8 cells, while the 4D8 antibody recognizes a unique striated fiber (of unknown composition) in the cytoplasm of macrogamonts. Compounds experimentally confirmed to target specific stages of the lifecycle, as evidenced by imaging studies, are presented.

This review focuses on our current understanding of the *Cryptosporidium* lifecycle, gained through genetics, advanced microscopy, and transcriptomic approaches. We also highlight a few anticryptosporidial compounds that demonstrate efficacy *in vitro* and/or in animal infection models and inhibit different stages of parasite development, as evidenced by imaging studies. Figure 1 illustrates the various stages of the *Cryptosporidium* lifecycle and their timing, along with a small panel of antibodies and stains used to distinguish parasite stages.

## *Cryptosporidium* developmental lifecycle: highly timed and coordinated

The lifecycle of *Cryptosporidium* begins when the thick-walled oocyst is ingested by a human or animal host. The oocyst excysts to release the four haploid sporozoites into the lumen of the small intestine. Changes in pH, bile salts, and the action of proteases have been reported as triggers for the induction of excystation [23]. These sporozoites have gliding motility and penetrate the mucus layer to attach to the intestinal epithelial cells. Upon contact with the cell, the parasite induces host actin polymerization and massive host cell remodeling through proteins it secretes into the host cell [24–27]. This host cell remodeling results in the microvillus membrane encapsulating the sporozoite, but the parasite never fully enters the host cell cytoplasm. Thus, the parasite establishes a unique epicellular (‘intracellular but extracytoplasmic’) niche for itself, where it resides and develops within a parasitophorous vacuole, yet remains separated from the host cell cytoplasm [28]. This unique epicellular localization is maintained throughout the parasite’s developmental cycle. Electron and fluorescence microscopy studies have shown that multiple structures separate the parasite from the host cell and restrict it to this extracytoplasmic location. These include a feeder organelle, a ring-like tight junction structure, an electron-dense band, and finally the actin pedestal at the base of the host–parasite interface [25,26]. After invasion, the sporozoite differentiates into a trophozoite, which then undergoes development, followed by three cycles of asexual replication to produce meronts (merogony). Merozoites released from the meront after the third replication cycle take an obligatory route to sexual development, differentiating into male and female sexual stages [22,29]. The male gametes (microgametes) released from the male gamont (microgamont) fertilize the female gamont (macrogamont). The resulting zygote (diploid) undergoes meiosis and sporogony to form an oocyst with four sporozoites. The oocyst is shed in the host’s feces or can initiate an autoinfection cycle within the same host (Figure 1).

As illustrated in Figure 1, the lifecycle of *Cryptosporidium* is highly programmed, proceeds in a coordinated manner, and takes 72 hours to complete. Infection of HCT-8 cells with fluorescent *C. parvum* reporter strains and time-lapse microscopy have revealed the dynamics and progression of the lifecycle [22,29]. A single cycle of asexual replication takes approximately 12 hours, and the parasite undergoes three rounds of replication. Live imaging of meronts has demonstrated that after the third round of asexual replication (36 hours), the merozoites released from a mature 8-nucleated meront are sexually committed and can develop into either male or female gamonts. The sex ratio is estimated to be twice the number of females to male gamonts for merozoites egressing from a single meront. Thus, there is no type-II meront, which was previously thought to be the precursor to the sexual stages [29]. Sexual development continues, with most parasites at 48 hours being male or female, and over 80% of sexual stages are seen at 72 hours post-infection [29].

In the following sections, we provide a concise overview of each stage of the *Cryptosporidium* life cycle and discuss the functions of selected proteins that have been functionally characterized or experimentally localized at different parasite stages. Table 1 summarizes the roles of the proteins discussed throughout the article. Additionally, we highlight potent compounds that have been shown to target various stages of parasite development.

### Sporozoite: attachment and invasion of the host cell

Live-imaging studies have shown that host cell invasion by the sporozoite is rapid. Sporozoite attachment occurs within a few seconds of contact, followed by bending and straightening (1–2 minutes) for its complete encapsulation by the host membrane, and transforms into a trophozoite by 8 minutes [30]. The sporozoite releases secretory proteins for attachment and invasion of the host cell, but only a few have been experimentally characterized. Recently, spatial proteomics of the sporozoite has identified 154 secretory proteins that are stored in four secretory organelles: micronemes, rhoptries, dense granules, and small granules (an organelle unique to this parasite), but only a handful have been functionally characterized to date [31].

Regarding micronemal proteins, the localization and functional roles of a few *Cryptosporidium* proteins involved in parasite motility, attachment, and invasion have been described. These include the thrombospondin repeat-containing (TSP) family of proteins that are c-mannosylated and contribute to parasite attachment and invasion [32]. The *C. parvum* TSP1 (TRAP-C1) localizes to the apical end and surface of sporozoites, while TSP8 (MIC1) and TSP4 localize to micronemes, likely playing roles in gliding motility and adhesion [32–35]. Interestingly, *C. parvum* TSP4 is trafficked along two unique central microtubules and is secreted during sporozoite excystation, gliding, and invasion. Selective kinesin-5 inhibitors have been shown to block the secretion and transport of TSP4 [34]. Mucin-like glycoproteins such as GP60 (which is proteolytically cleaved into GP40/GP15), GP900, CP23, MUC1-MUC7, and apical complex glycoproteins AGP1 and AGP2 have been shown to play roles in parasite motility, attachment, and invasion [36–43]. Some of the immunogenic mucin-like glycoproteins (namely GP40, GP15, CP23, GP900, and MUC8) have been proposed as vaccine candidates because of their roles in parasite attachment and invasion, as well as the antibody responses they elicit, which are linked to protection against reinfection in both humans and animals [44,45]. Recently, the European Medicines Agency approved a recombinant vaccine (Bovilis Cryptium^®^) based on *C. parvum* GP40 for use in neonatal calves to provide protection against cryptosporidiosis. Inoculating pregnant cows with recombinant GP40 protein produces antibodies against this protein that are passively transferred through colostrum to newborn calves, and this has been shown to reduce the severity and duration of diarrhea, although it does not fully prevent infection [45]. Additionally, a *C. parvum* rhomboid protease, ROM1, which functions in invasion and proteostasis of the parasitophorous vacuole membrane and feeder organelle, along with four proteins (encoded by cgd3_980, cgd1_3550, cgd1_3680, and cgd2_1590 genes), has also been localized to the micronemes using immunoelectron microscopy [46].

Regarding rhoptry proteins, one rhoptry neck protein (PRP1), a homolog of *Toxoplasma gondii* RON1, localizes to the organelle in sporozoites, merozoites, and at the parasite–host cell interface in developing trophozoites [47]. The *C. parvum* rhoptry bulb protein ROP1 is injected into the host cell during invasion and interacts with the host cytoskeletal modulator LMO7. Genetic ablation of ROP1 has been shown to reduce infectivity in immunocompromised mice [30]. Additionally, eight rhoptry bulb proteins (ROPs) have been identified at the apical end of sporozoites that are secreted during invasion, but their functions remain uncharacterized [30]. Regarding dense granules, six proteins (DG1 to DG6) have been localized to this organelle; however, their molecular functions in host–parasite interactions are not known [31,48]. Recently, deletion of DG6 has been shown to reduce parasite virulence *in vivo* [48]. Concerning small granule proteins, SG1, SG2, SKSR1, and MVP1 have been localized to this organelle [31,49,50]. Genetic deletion of SKSR1 or a base insertion to change the reading frame has been shown to reduce parasite virulence [49]. MVP1 is an exported virulence factor that interacts with host EBP50 and CDC42 to drive elongation of intestinal microvilli during infection [50].

Overall, the mechanism by which *Cryptosporidium* invades to create its unique epicellular localization is particularly intriguing. The encapsulation of the parasite by host membranes likely serves as an adaptation that shields it from immune recognition and clearance. What other benefits the parasite derives from this unique epicellular positioning, and the molecular processes that prevent it from becoming fully intracellular, remain to be investigated. Further research on the role of *Cryptosporidium* secretory effector proteins in host cell manipulation would enhance our understanding of the molecular mechanisms of attachment and invasion.

### Trophozoite: development and nutrient uptake

The trophozoite undergoes growth and development before DNA replication occurs. The feeder organelle is fully formed in the mature trophozoite and is also observed in all intracellular stages. This organelle is an elaborate network of tubular and bursiform membranes that has been historically described for its role in the uptake of essential nutrients from the host cell that the parasite cannot synthesize on its own [51]. *Cryptosporidium* has a very streamlined metabolism and salvages many metabolites, including purines, pyrimidines, fatty acids, amino acids, glucose-6-phosphate, and reduced glutathione from the host cell [41,52–54]. Furthermore, the parasite lacks an apicoplast and mitochondrion organelles but has a mitochondrial remnant (mitosome), does not have the TCA cycle, and relies on glycolysis for energy production [55].

Given the heavy dependency of *Cryptosporidium* on the host cell for metabolites, it is not surprising that the compact 9.1 Mb parasite genome encodes an extensive repertoire of 152 transporters [56]. However, the localization and function of most of these transporters remain unknown. So far, only four *C. parvum* transporters have been localized to the feeder organelle. These include the *C. parvum* ATP-binding cassette (ABC1) transporter and two glucose transporters, GT1 and GT2 [54,57]. Both GT1 and GT2 transport glucose-6-phosphate, whereas GT1 also transports glucose from the host cell [54]. Interestingly, a recent study has identified the role of the multidrug-resistant protein (CpMRP1), an ABC transporter, in exporting the toxic microbial metabolite deoxycholate to establish infection and contribute to virulence in mice [58]. This suggests that metabolites produced by the gut microbiota can restrict *Cryptosporidium* growth, but the parasite has evolved mechanisms to export microbial metabolites to thrive in the intestinal environment.

Regarding the inhibition of the trophozoite stage, BRD7929, a bicyclic azetidine, has been demonstrated to block its development. EdU (5-ethynyl-2’-deoxyuridine) incorporation and imaging indicate that BRD7929 treatment stalls the parasite at the early trophozoite stage, resulting in an expanded feeder organelle and a lack of DNA replication [59]. BRD7929 targets the phenylalanyl tRNA synthetase (PheRS), effectively killing *Cryptosporidium in vitro* and in immunocompromised mice [18]. In fact, aminoacyl-tRNA synthetases (aaRS) are conserved biological targets across protozoan parasites, and compounds targeting PheRS, methionyl tRNA synthetase (MetRS), as well as lysyl tRNA synthetase (KRS) in *Plasmodium* or *Trypanosoma*, have been optimized for *Cryptosporidium* and shown to kill the parasite both *in vitro* and *in vivo* [18,60–62].

Regarding the feeder organelle, many questions remain in the field. How the feeder organelle forms and its boundaries are still unclear. Is it open to the host cytoplasm? Which metabolites are transported to the parasite? Additionally, elucidating the role of transporters involved in compound efflux may have significant implications for understanding the development of resistance to new anticryptosporidial drugs.

### Asexual proliferation (merogony)

During a single asexual proliferation cycle, the trophozoite undergoes three nuclear divisions to form an 8-nucleated meront. Transcriptomic data show that DNA replication and ribosomal genes are expressed during the meront growth phase, followed by expression of membrane and apical structure components, and finally the secretion machinery, which forms all four apical organelles for merozoite invasion [63]. Merozoites that egress from the meront are motile and invade new enterocytes [22,29]. Although the molecular mechanisms governing meront development and merozoite egress are not well understood, calcium signaling is reported to play an important role in this process [17]. The calcium-dependent protein kinase 1 (CDPK1) is a leading drug target, and selective bumped kinase inhibitors (BKI-1294, BKI-1708) have shown efficacy in killing *Cryptosporidium* in both immunocompromised mice and calf infection models [64–66]. CDPK1 is essential for parasite survival and is expressed in trophozoites, 8-nucleated meronts, and merozoites but not in the 4-nucleated meronts, indicating it is cell-cycle regulated [17]. Conditional knockdown of CDPK1 has been demonstrated to cause defects in merozoite development [17]. Notably, the promising clinical candidate, EDI048, a soft-drug that inhibits parasite PI(4)K (phosphatidylinositol-4-OH kinase), has been shown to block membrane development in merozoites, thus stalling them at the meront stage [67]. Additionally, the *C. parvum* cyclic GMP-dependent protein kinase G (PKG) and aspartyl protease 2 (ASP2) have been reported to play roles in merozoite egress [68,69]. Thus, targeting merozoite development and egress provides attractive targets for drug development since they can prevent both parasite proliferation and entry into the sexual cycle.

Together, these transcriptomics, cell biology, and drug discovery studies have enhanced our understanding of merozoite development and egress. However, many questions still need to be investigated. For example, the mechanisms by which *Cryptosporidium* counts three rounds of replication during merogony remain unclear. The entry of merozoites into the sexual cycle after the third replication is particularly intriguing. Environmental cues sensed by the parasite and transcriptional regulation might play a role in this highly programmed process, but further research is necessary to uncover these mechanisms.

### Microgamont development and egress of male gametes

Microgamonts develop and undergo four rounds of nuclear division to produce 16 microgametes. Early microgamonts (~42 hours) have their eight nuclei arranged in a compact, rosette-shaped pattern, and as they develop, the nuclei become more rounded, ultimately resulting in bullet-shaped nuclei in the 16 non-flagellated male gametes [22,70]. The *C. parvum* single-cell transcriptomics atlas has mapped 389 genes to three clusters (early, mid, and late males), with 51 genes that are exclusively expressed only at this stage [63]. These 51 male-exclusive genes encode transcription factors, signaling molecules, cell cycle regulators, proteases, secretory proteins, and membrane proteins. To date, only two transcription factors have been functionally validated in male development. The Myb-M transcription factor is the earliest determinant of male fate, and its conditional genetic deletion results in the complete loss of male gametes *in vitro* and the absence of oocyst shedding *in vivo* [63]. The Apetala-2 transcription factor, AP2-M, is expressed exclusively in microgamonts during development and has been reported to be essential for parasite survival [71].

Furthermore, the *C. parvum* calcium-dependent protein kinase-5 (CDPK5) has been shown to play a role in the egress of male gametes and in parasite virulence, with substrates of this kinase identified using phosphoproteomics [70]. Additionally, ASP2, which is important for merozoite egress, has also been shown to be necessary for male gamete egress [68].

Two other male-specific genes that have been localized to male gametes encode for hapless 2 protein (HAP2) and the secretory family protein GGC1 [22,63]. HAP2 localizes to the apical pole of microgametes, while GGC1 localizes to a bar-like structure at the apex. This suggests that HAP2 and GGC1 may be components of the adhesion apparatus or fertilization machinery required for the fusion of male gametes with the female gamont.

Many questions about male gametogenesis and fertilization remain unanswered. Male gametes released from microgamonts are immotile, yet they somehow travel toward the macrogamont; how this occurs remains a mystery. Moreover, the molecular mechanisms underlying the adhesion of the male gamete to the female gamont for fertilization are yet to be discovered.

### Macrogamont development

The single-nucleated macrogamont develops within the parasitophorous vacuole and does not undergo nuclear division or egress, but increases its size during development. Electron microscopy studies have reported that the macrogamont synthesizes the components of the oocyst wall, the wall-forming bodies 1 and 2, as well as amylopectin granules [72,73]. These amylopectin stores are degraded by the parasite glycogen phosphorylase, which releases phosphorylated glucose required for glycolysis and ATP generation [54]. Bulk and single-cell RNA sequencing of fluorescent reporter parasites *in vitro* and *in vivo* have identified genes that are highly expressed in the females, and these encode for oocyst wall synthesis, energy storage (amylopectin and trehalose metabolism), meiosis and DNA repair, glycosyltransferases, proteases, polyketide, and fatty acid synthesis [22,63]. Out of the 773 genes enriched in the female transcriptome, 187 genes are expressed exclusively at this stage.

Few proteins have been genetically characterized for their roles in macrogamont development. Conditional deletion of the essential female-specific AP2 transcription factor (AP2-F) has been shown to downregulate expression of genes encoding the DNA meiotic recombinase (DMC1), NIMA kinase 5, DNA polymerase, and crystalloid body proteins (an organelle of unknown function found in sporozoite), and significantly reduce oocyst shedding in IFN-γ KO mice [71]. Another protein reported to play a role in macrogamont development is the insulinase-like protease 1 (INS1), which belongs to the M16 family of metallopeptidases. INS1 localizes to small secretory vesicles near wall-forming bodies in the macrogamont, and genetic deletion of INS1, or its replacement with an active-site mutant, results in reduced macrogamont numbers *in vitro* and decreased oocyst shedding *in vivo* [74].

Two female-specific antibodies, DMC1 and 4D8, have proven useful for identifying compounds that specifically target macrogamont development. A DMC1 antibody-based high-content imaging assay identified nine potent small molecule compounds from a drug repurposing library that inhibited macrogamont differentiation [75]. Moreover, the piperazine-based inhibitor, MMV665917, a highly efficacious anticryptosporidial compound, was found to inhibit macrogamont development in DMC1 and 4D8-based imaging assays [59,76,77]. Interestingly, the fiber structure in the cytoplasm of macrogamonts stained with the 4D8 antibody was much shorter after treatment with this compound, although the composition of this fiber is not known [59].

Together, these studies show that the macrogamont is transcriptionally and translationally active and prepares for oocyst formation. Further research is required to understand the molecular mechanisms that regulate macrogamont maturation, as well as the formation of amylopectin granules and oocyst wall components.

### Oocyst: forming the protective wall structure

The oocyst has a multilayered structure that protects the sporozoites inside and includes a glycocalyx, inner layers of acid-fast lipids, and an innermost layer of nine cysteine- and histidine-rich oocyst wall proteins (COWPs) [73,78–80]. COWP1 and COWP8 are expressed in the oocyst wall, as shown through immunolocalization and immunofluorescence studies, but COWP8 does not contribute to the strength of the oocyst wall nor is it required for transmission [73,80,81]. Interestingly, COWP2, COWP3, and COWP4 localize to the suture, which is the region where the sporozoite exits the oocyst [81]. Historically, thin-walled oocysts that easily excyst within the same host have been linked to the autoinfection cycle, while thick-walled oocysts have been associated with transmission [72].

Understanding how the oocyst wall is constructed would elucidate the oocyst’s resistance to disinfection and inform the development of compounds that target key wall components to reduce environmental contamination.

## Concluding remarks

Overall, new insights into the *Cryptosporidium* lifecycle are enhancing our fundamental understanding of parasite biology and helping identify vulnerabilities that can be targeted for drug and vaccine development. Insights into the host–parasite interactions, characterization of transporter function, and identification of critical metabolites that the parasite scavenges from the host will open new avenues for both parasite- and host-targeted therapies. Future research on the molecular mechanisms underlying the development of sexual stages and the fertilization process will improve our understanding of the parasite’s lifecycle. Thus, targeting either the initial stages of infection, merogony, or the sexual development cycle to prevent transmission are promising strategies to combat cryptosporidiosis and reduce its significant public health burden.

## Figures and Tables

**Table 1:** *Cryptosporidium parvum* proteins and their roles at different parasite stages.

Article section	Protein	Gene ID	Function^a^	References
Sporozoite	TSP1	cgd1_3500	Attachment/invasion	[32,35]
Sporozoite	TSP4	cgd8_150	Attachment/invasion	[34]
Sporozoite	TSP8	cgd6_780	Attachment/invasion	[32,33]
Sporozoite	GP60	cgd6_1080	Attachment/invasion	[36,37,42]
Sporozoite	GP900	cgd7_4020	Attachment/invasion	[38]
Sporozoite	CP23	cgd4_3620	Attachment/invasion	[31,41]
Sporozoite	MUC1-MUC7	cgd2_390–450	Attachment/invasion	[40,43]
Sporozoite	AGP1	cgd4_32	Attachment/invasion	[39]
Sporozoite	AGP2	cgd7_4330	Attachment/invasion	[39]
Sporozoite	ROM1	cgd3_980	Attachment/invasion	[46]
Sporozoite	PRP1	cgd8_2540	Invasion	[47]
Sporozoite	ROP1	cgd3_1770	Host remodeling; invasion	[30]
Sporozoite	SKSR1	cgd1_140	Virulence factor	[49]
Sporozoite	MVP1	cgd6_40	Microvilli elongation	[50]
Trophozoite	ABC1	cgd1_700	Transporter	[54,57]
Trophozoite	GT1	cgd3_4070	Transporter	[54]
Trophozoite	GT2	cgd4_2870	Transporter	[54]
Trophozoite	MRP1	cgd7_4520	Transporter (efflux)	[58]
Merogony	CDPK1	cgd3_920	Merozoite development	[17]
Merogony	P1(4)K	cgd8_4500	Merozoite development	[67]
Merogony	PKG	cgd8_750	Merozoite egress	[69]
Merogony and Microgamont	ASP2	cgd1_3690	Merozoite egress and Microgamete egress	[68]
Microgamont	Myb-M	cgd6_2250	Microgamont commitment	[63]
Microgamont	AP2-M	cgd6_2670	Microgamont development	[63,71]
Microgamont	CDPK5	cgd2_1300	Microgamete egress	[70]
Microgamont	HAP2	cgd8_2220	Gamete fusion	[22,63]
Microgamont	GGC1	cgd7_5500	Gamete fusion	[22,63]
Microgamont	AP2-F	cgd4_1110	Macrogamont development	[63,71]
Macrogamont	INS1	cgd1_1680	Macrogamont development	[74]
Macrogamont	DMC1	cgd7_1690	Macrogamont development	[75,77]
Macrogamont and Oocyst	COWP1	cgd6_2090	Macrogamont development and Oocyst formation	[73,80]
Oocyst	COWP2	cgd7_1800	Oocyst wall formation	[80,81]
Oocyst	COWP3	cgd4_670	Oocyst wall formation	[80,81]
Oocyst	COWP4	cgd8_3350	Oocyst wall formation	[80,81]
Oocyst	COWP8	cgd6_200	Oocyst wall formation	[80,81]

a Characterized or putative function based on functional studies or localization.

## Data Availability

No data were used for the research described in the article.
